# Influenza vaccine in pregnant women: immunization coverage and associated factors

**DOI:** 10.1590/S1679-45082013000100010

**Published:** 2013

**Authors:** Renato de Ávila Kfouri, Rosana Richtmann

**Affiliations:** 1 Hospital e Maternidade Santa Joana, São Paulo, SP, Brazil.

**Keywords:** Influenza vaccines, Pregnancy complications, infectious, Influenza, human/prevention & control, Infant, newborn, Maternal-fetal exchange, Immunization programs

## Abstract

**Objectives:**

To describe the immunization coverage of the influenza vaccine for pregnant women, and factors associated to vaccination compliance.

**Methods:**

This is a prospective, descriptive study including 300 women who had just given birth at Hospital and Maternity Santa Joana in Sao Paulo, Brazil. Data were collected through a pre-tested questionnaire applied by a trained evaluator during October 2010.

**Results:**

The mean age of mothers was 30.5 years; 231 (77%) were married; 164 (54.7%) were primigravidas; 192 (64%) had higher education; and 240 (80%) were employed. During the prenatal period, 234 (78%) received information about the influenza vaccine and 287 (95.7%) were immunized; 210 (73.2%) women knew about neonatal protection achieved through maternal vaccination. The factors associated with maternal acceptance of the vaccine were government campaign (133; 44.3%), and medical recommendation during prenatal visits (163; 54.3%). A total of 13 pregnant women refused vaccination for the following reasons: neglect (4), lack of time (4), lack of recommendation from their physician (3) or contraindication by physician (2), but 69.2% of them would have accepted immunization had they been informed about neonatal protection.

**Conclusions:**

The fear of a pandemic and the public vaccination campaign had an important impact on the high immunization coverage for influenza on pregnant women. Medical recommendation and the government campaign were the main reasons for vaccine compliance.

## RESUMO

**Objetivo:**

Descrever a cobertura vacinal de gestantes para influenza e fatores associados à recusa ou à aceitação da vacina.

**Métodos:**

Estudo descritivo, prospectivo, que incluiu 300 puérperas atendidas no Hospital e Maternidade Santa Joana (SP). Os dados foram coletados em outubro de 2010, por meio de questionário pré-testado, aplicado por profissional treinado.

**Resultados:**

A média de idade das mães foi 30,5 anos; 231 (77%) eram casadas; 164 (54,7%) primigestas; 192 (64%) tinham nível superior de escolaridade; e 240 (80%) estavam empregadas. Durante o pré-natal, 234 (78%) receberam informação sobre a vacina contra influenza e 287 (95,7%) foram imunizadas; 210 (73,2%) mulheres tinham conhecimento sobre a proteção neonatal pela vacinação materna. Fatores associados à aceitação foram: campanha do governo (133; 44,3%) e recomendação médica durante o pré-natal (163; 54,3%). Entre as 13 grávidas que recusaram a vacinação, as razões foram: negligência (4), falta de tempo (4), falta de indicação médica (3) ou contraindicação pelo médico (2), mas 69,2% teriam sido vacinadas se tivessem sido informadas sobre a proteção neonatal.

**Conclusão:**

O receio da pandemia e a campanha pública tiveram importante impacto na elevada cobertura vacinal para influenza em gestantes. Recomendação médica e campanha do governo foram os principais motivos de aceitação da vacina.

## INTRODUCTION

Seasonal Influenza affects roughly 10% of the world population on a yearly basis. There are an estimated 3 to 5 million cases of severe disease associated with the virus, and approximately 250,000 to 500,000 deaths yearly due to the disease, worldwide. Specific populations, such as the elderly, immunocompromised individuals, children and pregnant women, are more susceptible to severe disease, as well as influenza-associated complications, hospitalization and death^(1)^.

Influenza vaccination is widely recommended for these individuals and, in the case of pregnant women, is capable of protecting the mother, as well as indirectly the newborn (NB), who cannot be vaccinated until 6 months of age. Newborn protection occurs through passive transfer of antibodies^(2)^. Puck et al. described the passive transfer of antibodies from mother to fetus, and to the newborn^(3)^.

In a case-control study, Benowitz et al. demonstrated the effectiveness of vaccination of pregnant women to prevent hospitalization of children younger than 6 months due to influenza, in 91.5% of cases^(4)^. In a prospective cohort study, Eick et al. observed that infants had a drop by 41% in risk of influenza infection, confirmed by laboratory tests, in the first 6 months of life if their mothers had been vaccinated during pregnancy^(5)^.

The American Academy of Pediatrics recommends the inactivated virus vaccine to all in close proximity to infants who are at a higher risk of infection, including parents and caregivers^(6)^. The literature indicates that the influenza vaccination compliance rate among parents is very low. The influenza vaccination compliance rate in the general population is approximately 25 to 32%. A study designed to establish influenza compliance rates among parents, and to determine the reasons for non compliance, revealed that although 92% of parents had the intention of being immunized, only 32.6% of them had actual access to the vaccine^(7)^. Difficult access to vaccine was the most reported reason for non-vaccination. Shah et al. observed an influenza vaccination compliance rate among parents of 23.2% in an epidemiologic study evaluating parents of newborns in a neonatal intensive care unit (NICU). All parents in this study agreed to take the vaccine after learning about the importance of indirect protection to their offspring through their immunization^(8)^.

A Canadian study performed at the time of a vaccination campaign during the 2009-2010 pandemics demonstrated the importance of the internet as a tool to disseminate information and to foster vaccination compliance^(9)^.

As from April 2010, in Brazil, the monovalent vaccine against the pandemic A (H1N1) influenza strain has been available to pregnant women, regardless of the stage of pregnancy and free of charge, through the public health system. This measure was based on epidemiological data obtained during the 2009 influenza pandemic. Pregnant women were at increased risk of morbidity and mortality in the country, with 189 confirmed deaths in 2009 alone^(10)^.

## OBJECTIVE

To describe the influenza immunization coverage in pregnant women and the factors associated to refusal or acceptance of the vaccine.

## METHODS

In this prospective and descriptive epidemiological study, a total of 300 women who had recently given birth answered an epidemiological questionnaire during their three-day post-partum stay in the maternity ward of Hospital e Maternidade Santa Joana, in Sao Paulo (SP). At this private hospital, an average of 1000 babies are delivered monthly, 91% of which via Cesarean section. Women who had just given birth were selected randomly (convenience sample).

Demographic data, schooling level, parity, profession, vaccination history, infuenza immunization coverage during pregnancy, reasons for compliance or noncompliance with influenza vaccine, and knowledge about indirect protection of the newborn were recorded. Data collection was performed during from October 1^st^ to 31^st^, 2010, by a single trained evaluator, after signature of the informed consent form. Data were organized in Excel spreadsheets for later statistical analysis.

### Statistical analysis

For statistical analysis the χ^2^ test and/or the Fisher test were used for quantitative variables, and the Student's *t* test was used for numerical variables, with p<0.05.

## RESULTS

Demographical characteristics of the sample population are summarized on table 1. The patient's mean age was 30.5 years.

**Table 1:** Demographic data (n=300)

Population data	n (%)
Married	231 (77.0)
Primipara	164 (54.7)
Higher education (schooling)	192 (64.0)
Employed	240 (80.0)

There were no significant differences between mothers who received the vaccine and those who did not. The majority (234; 78%) of the 300 women who were interviewed were informed about vaccination during the prenatal period, and 287 (95.7%) of them received the influenza vaccine during pregnancy, and 210 (73.2%) knew about neonatal protection. The 13 (4.3%) women who were not vaccinated were not aware of the fact that the vaccine would protect the newborn. Table 2 summarizes the characteristics of women who were immunized against infuenza and of those who were not. The influenza vaccine was recommended by the government campaign, or by the gynecologist-obstetrician for 133 (44.3%) and 163 (54.3%) women, respectively. Twenty (6.6%) out of the 300 women interviewed in this study were healthcare professionals, all of whom received the influenza vaccine. Table 3 shows vaccination compliance rates and knowledge about neonatal protection. The vaccine was given to 95% of the pregnant women (287/300) and 70% (210/300) were aware of neonatal protection. While only 27% (77/287) of the women who received the vaccine did not know about neonatal protection, all of the 13 women who refused vaccination were unaware that it would protect the newborn. Table 4 shows that, among the 13 women who refused the vaccine, 9 (69.2%) would have accepted vaccination during gestation had they been informed about neonatal protection, and 11 (84.6%) would have taken the vaccine immediately after birth.

**Table 2:** Characteristics of pregnant women who received or not vaccine against influenza

Pregnant women	Yes (n)	No (n)	p value
Received vaccine against influenza	287	13	
Primigravida	156	8	0.778
Married	222	9	0.505
Higher education (schooling)	184	8	0.915
Employed	231	9	0.300
Mean age (years)	30.6	29.0	0.428
Was aware about vaccine protecting the NB	210	0	0.000011*

* significant.

NB: newborn.

**Table 3:** Acceptance of vaccination and knowledge about neonatal protection

Unawareness of vaccine protection	Vaccine status		Total	p value
	Received	Refused		
Yes	77	13	90	
No	210	0	210	0.000001*
Total	287	13	300	

* significant.

**Table 4:** Reasons given by pregnant women for refusing vaccination

Reasons for refusing	n (%)
Neglect	4 (30.7)
Lack of time	4 (30.7)
Lack of recommendation from physician	3 (23.0)
Contraindicated by physician	2 (15.4)

## DISCUSSION

In 2009, a novel strain of the Influenza A virus (subtype H1N1) spread across the globe causing a pandemic outbreak (*avian influenza*). The World Health Organization estimates that the avian flu infected around 30% of the world population^(11)^. Approximately 1,700 deaths due to avian flu were notified in Brazil in 2009. Among the populations at a higher risk of developing a severe form of the disease and related complications are children, healthcare professionals, pregnant women and young adults. The 2010 government campaign for influenza vaccination in Brazil targeted these specific populations^(12)^. Differently from what is observed during seasonal influenza epidemics, infection of elderly people with the new H1N1 strain did not increase morbidity or mortality in this group.

Pregnant women are at a higher risk of developing influenza-related complications and of being hospitalized due to the virus, especially during the third trimester^(13,14)^. Up until 2010, the Brazilian National Vaccination Program (PNI, acronym in Portuguese) did not offer free influenza vaccines, in spite of the strong recommendation made by the Brazilian Immunization Society for the vaccination of pregnant women, as routinely performed in many other countries, such as the United States, Canada and in the majority of European countries^(15,16)^.

Many studies demonstrate the safety of the influenza vaccine for pregnant women^(17,18)^. It had also been reported in the literature that immunization coverage has been low among pregnant women in the past few years. Lu et al. estimated an average 14% immunization coverage among pregnant women in the United States between 1989 and 2005^(19)^. Lau et al. interviewed 568 pregnant women in Hong Kong and found that while 85.4% of them knew about the influenza vaccine, only 21.3% were actually vaccinated. Multivariate analysis demonstrated that vaccination compliance was higher when the vaccine was recommended by a healthcare professional^(20)^. A high immunization coverage rate among pregnant women was demonstrated in this study. In a report by the Centers for Disease Control and Prevention (CDC) the influenza vaccination coverage rates among pregnant women from ten different states across the United States were estimated for the 2009 – 2010 period. Coverage rates were 50.7% for seasonal influenza and 40.6% for pandemic influenza A/H1N1 in 2009, and were significantly higher than those recorded for previous years. Additionally, women to whom the vaccine had been recommended by a healthcare professional were significantly more likely to get vaccinated against seasonal influenza (RR=3.3) and against influenza A/H1N1/2009 (RR=10.1)^(21)^. A study carried out in Texas, in the United States, showed that influenza vaccination coverage among pregnant women and health professionals increased from 2.5 to 37.4%, and from 36 to 64% during the 2003-2004 and the 2008-2009 seasons, respectively, after implementation of specific strategies^(22)^.

The transfer of maternal antibodies through the placenta and subsequently through breast milk, is capable of protecting breastfeeding babies against the influenza virus for the first 6 months of life, a period during which the newborn cannot yet be vaccinated^(23)^. Benowitz et al., in a case control study, demonstrated that 91.5% of the infants born to immunized mothers were protected against hospitalization due to influenza during the 6 first months of life (95% confidence interval – CI95%: 61.7-98.1%)^(4)^. Eick et al., in a prospective, observational cohort study, showed that breastfeeding infants born to mothers who had been immunized during pregnancy had 41% less risk of contracting influenza infection as confirmed by laboratory exams, and 39% less risk of being hospitalized due to acute respiratory illness. Children born from women who had been vaccinated against influenza also had significantly lower strain-specific antibody titers, as measured through a standard hemagglutinin inhibition assay, at the time of birth and at 2-3 months of age, when compared to children born to unvaccinated mothers^(5)^.

Brazilian epidemic surveillance data demonstrated a higher rate of complications and deaths among pregnant women in 2009, justifying the inclusion of this population group in the vaccination campaign. In 2010, two forms of the influenza vaccine were in use in Brazil: the monovalent A/H1N1 vaccine, utilized throughout the public health system, and the trivalent vaccine against the A/H1N1, A/H3N2 and B strains, available only through private health care^(24)^.

Despite the knowledge that vaccination during pregnancy protects the newborn, immunization coverage in pregnant women remains low. American epidemiologic surveillance data from the CDC estimate immunization coverage of 15.5% in this group^(25)^. In 2010, immunization coverage rates were high in general throughout Brazil, due to extensive media coverage, to the fear of a pandemic outbreak, and to vaccination campaigns^(26)^. A high vaccine coverage rate was observed in the present study, probably due to the same factors. All of the pregnant healthcare professionals were vaccinated. Only 13 out of the 300 women interviewed were not immunized against influenza, but 69.2% of those who were not vaccinated would have accepted the vaccine had they been informed about neonatal protection.

## CONCLUSIONS

Influenza vaccine coverage among pregnant women was high during a pandemic outbreak year due to effective vaccination campaigns. More than half of the pregnant women accepted the vaccine after medical recommendation. One third of the women that had higher education were unaware of the benefits of the vaccine in terms of neonatal protection. Knowledge of neonatal protection could increase the immunization coverage rates. Vaccination of mothers who have just given birth, during their stay in the maternity ward, may be a useful tool to further improve vaccination coverage. Sustained efforts towards disseminating awareness about the benefits of such strategies among healthcare professionals and of the general public may lead to high vaccination coverage rates among pregnant women.
